# Liposomes Loaded with Unsaponifiable Matter from *Amaranthus hypochondriacus* as a Source of Squalene and Carrying Soybean Lunasin Inhibited Melanoma Cells

**DOI:** 10.3390/nano11081960

**Published:** 2021-07-30

**Authors:** Erick Damian Castañeda-Reyes, Elvira Gonzalez de Mejia, Fred Joseph Eller, Mark A. Berhow, María de Jesús Perea-Flores, Gloria Dávila-Ortíz

**Affiliations:** 1 Department of Food Science and Human Nutrition, University of Illinois, Urbana-Champaign, IL 61801, USA; edreyes@illinois.edu; 2 Departamento de Ingeniería Bioquímica, Escuela Nacional de Ciencias Biológicas, Instituto Politécnico Nacional (IPN), Av. Wilfrido Massieu, esq. Miguel Stampa s/n, Zacatenco, Alcaldía Gustavo A. Madero, Ciudad de México 07738, Mexico; 3 Functional Foods Research, United States Department of Agriculture, National Center for Agricultural Utilization Research†, Midwest Area, Agricultural Research Service, 1815 N. Univ. St., Peoria, IL 61604, USA; fred.eller@usda.gov (F.J.E.); mark.berhow@usda.gov (M.A.B.); 4 Centro de Nanociencias y Micro y Nanotecnologías, Instituto Politécnico Nacional (IPN), Av. Luis Enrique Erro s/n, Unidad Profesional Adolfo López Mateos, Zacatenco, Alcaldía Gustavo A. Madero, Ciudad de México 07738, Mexico; mpereaf@ipn.mx

**Keywords:** *Amaranthus hypochondriacus*, amaranth unsaponifiable matter, liposomes, lunasin, melanoma, squalene, supercritical fluid extraction

## Abstract

*Amaranthus hypochondriacus* is a source of molecules with reported health benefits such as antioxidant activity and cancer prevention. The objective of this research was to optimize the conditions for preparing a liposome formulation using amaranth unsaponifiable matter as a source of squalene in order to minimize the particle size and to maximize the encapsulation efficiency of liposomes for carrying and delivering soybean lunasin into melanoma cell lines. Amaranth oil was extracted using supercritical dioxide carbon extraction (55.2 MPa pressure, 80 °C temperature, solvent (CO_2_)-to-feed (oil) ratio of 20). The extracted oil from amaranth was used to obtain the unsaponifiable enriched content of squalene, which was incorporated into liposomes. A Box–Behnken response surface methodology design was used to optimize the liposome formulation containing the unsaponifiable matter, once liposomes were optimized. Soybean lunasin was loaded into the liposomes and tested on A-375 and B16-F10 melanoma cells. The squalene concentration in the extracted oil was 36.64 ± 0.64 g/ 100 g of oil. The particle size in liposomes was between 115.8 and 163.1 nm; the squalene encapsulation efficiency ranged from 33.14% to 76.08%. The optimized liposome formulation contained 15.27 mg of phospholipids and 1.1 mg of unsaponifiable matter. Cell viability was affected by the liposome formulation with a half-maximum inhibitory concentration (IC_50_) equivalent to 225 μM in B16-F10 and 215 μM in A-375. The liposomes formulated with lunasin achieved 82.14 ± 3.34% lunasin encapsulation efficiency and improved efficacy by decreasing lunasin IC_50_ by 31.81% in B16-F10 and by 41.89% in A-375 compared with unencapsulated lunasin.

^†^ Mention of trade names or commercial products in this publication is solely for the purpose of providing specific information and does not imply recommendation or endorsement by the U.S. Department of Agriculture.

## 1. Introduction

Amaranth (*Amaranthus* spp.) is considered a pseudocereal because it neither belongs to the grass family nor contains gluten, which makes this seed attractive for people with celiac disease [1,2]. Amaranth seeds are a good source of bioactive compounds whose reported health benefits include antioxidant activity and the prevention of hypertension, lipid disorders, and some types of cancer [1,3]. The antioxidant activity is attributed to molecules located in the unsaponifiable matter, composed of squalene (SQ), tocopherols, sterols, and other minor compounds [1,4,5,6]. 

SQ is a triterpene cholesterol precursor composed of six isoprene units [7,8]. SQ can be synthesized in animals, microorganisms, and vegetables [5,9]. The richest SQ source is shark liver oil, representing up to 40% of the liver weight [9]. Since these animals are protected, plant sources should be explored [9,10]. In plants, SQ is synthesized through the mevalonate pathway from two molecules of farnesyl diphosphate in reactions catalyzed by squalene synthase [11]. SQ could be extracted from a plant with organic solvents; however, flammable organic solvents can be replaced by a nontoxic solvent like carbon dioxide (CO_2_) under supercritical conditions [4,10,12].

Supercritical fluid extraction (SFE) has been used for SQ-enriched oil extraction from amaranth seeds [2,4,13]. SFE has been described as a safe and environmentally friendly alternative for the extraction of oil [2,14,15]. The most commonly used solvent for this technique is CO_2_, which is a nontoxic, low-cost, and nonpolar effective solvent for plant lipid extraction [12,16]. The use of SFE is recognized as an effective technique from the laboratory to the industrial scale [17]. SFE parameters (pressure, temperature, time, and flow rate) can be adjusted to increase the oil yield or the concentration of certain compounds such as SQ [12,15]. 

SQ has been used as a vaccine adjuvant and drug delivery emulsion since it can enhance effects and biocompatibility. An example of squalene as an adjuvant is the commercial nanoemulsion MF59 [18]. Other nanoparticles that can include squalene are liposomes. Liposomes are small spherical vesicles composed of one or more phospholipid bilayers, first introduced in the mid-nineteenth century to mimic the cell membrane [19,20]. Due to their amphipathic nature, liposomes are able to encapsulate, carry, and deliver into cells polar, nonpolar, and amphiphilic substances [21]. Furthermore, they favor the stabilization of the encapsulated compounds and improve the delivery efficiency and bioavailability of molecules [22,23]. Therefore, liposomes enhance the solubilization of hydrophobic molecules [24]. Some advantages of liposomes are the prevention of drug metabolization before reaching target tissues/cells, a decrease in healthy cells’ exposure to drugs, increased blood circulation, and protection against naturally occurring phenomena such as enzymatic degradation [25].

Liposomes have been successfully prepared and tested as amphipathic transporters of bioactive compounds for melanoma treatment. Melanoma is a type of skin cancer that has a five-year survival rate of 15% and 90% mortality [21,26]. 

Several reports have described the effects of different food-related bioactive compounds with antimelanoma activity, such as resveratrol [27,28], quercetin [29], and lunasin [30,31]. Lunasin is a peptide isolated from soybeans. Three domains have been identified as involved in lunasin’s anticancer activity: an Arg-Gly-Asp (RGD) region that allows for lunasin internalization; a polyaspartic region that binds histones H3 and H4, avoiding acetylation; and a helical chromatin-binding region [32]. Lunasin has been proven to be effective against melanoma [30,32,33].

The objective of this research was to optimize the conditions to prepare a liposome formulation based on amaranth unsaponifiable matter as a source of squalene in order to minimize particle size and maximize squalene encapsulation efficiency for delivering lunasin into melanoma cell lines B16-F-10 and A-375.

## 2. Materials and Methods

### 2.1. Materials

Amaranth seeds (*Amaranthus hypochondriacus*) of the Nutrisol variety were harvested in 2018 and donated by Mr. Everardo Lovera, president of the Maize Producers Federation of Mexico State, Mexico. 

The following materials were purchased from Sigma-Aldrich (St. Louis, MO, USA): squalene, ≥98% purity; squalane, ≥95% purity; 1,2-dioleoyl-sn-glycero-3-phosphocholine (DOPC); 1,2-dioleoyl-sn-glycero-3-phospho-rac-(1-glycerol) sodium salt (DOPA) and cholesterol, ≥99% purity. Boron trifluoride (BF_3_) (14% in methanol) was purchased from Alltech, Inc. (Deerfield, IL, USA) C8-C30 Fatty acid methyl ester (FAME) standards and triundecanoin were purchased from Nu-Check Prep (Elysian, MN, USA). Holey carbon grids for transmission electron microscopy (TEM) were purchased from Structure Probe, Inc. (SPI, West Chester, PA, USA). Cell Counting Kit-8 (CCK8) was acquired from Abcam (Cambridge, MA, USA). Amicon Ultra centrifugal filters (50 kDa cutoff) were purchased from Millipore (Burlington, MA, USA). Detergent-compatible (DC) protein assay was purchased from Bio-Rad (Hercules, CA, USA).

### 2.2. Methodology 

#### 2.2.1. Amaranth oil Supercritical Fluid Extraction (SFE) and Squalene Determination in Oil Using Supercritical Fluid Chromatography (SFC) 

The amaranth seed was ground for 1 min in an electric dry food grinder (model MX-228, Varco, Inc., Bellville, NJ, USA). Extraction of amaranth oil followed the methodology described by King et al. [34]. Briefly, 5 g of the ground material was placed in a Spe-ed supercritical fluid extractor from Applied Separations (Allentown, PA, USA). Two different preliminary extraction conditions were tested (pressure of 27.6 MPa at 40 °C and 55.2 MPa at 80 °C), and the combination of the conditions giving the highest yield was used for the extraction. Both procedures had a solvent-to-feed ratio of 20. Based on the observed yields, the 55.2 MPa at 80 °C extraction condition was chosen.

The composition of the amaranth SFE extracts was determined by SFC, using a Series 4000 SFC chromatograph (Selerity Technologies, Inc., Salt Lake City, UT, USA) equipped with a flame ionization detector (FID) held at 350 °C. Welding-grade carbon dioxide was used as the carrier fluid. An SB-Methyl-100 capillary column (5 m × 50 µm i.d., 0.25 µm film thickness) (Selerity Technologies, Inc., Salt Lake City, UT, USA) was used as explained by Eller et al. [35], with some modifications. A program of 100 °C isothermal, 10.1 MPa hold for 5 min, and a ramp of 1.5 MPa/min to 31.4 MPa was followed. A solution containing ca. 5 mg/mL was injected into the SFC (500 nL loop) with an injection duration of 0.5 s, and the relative amounts were determined from the FID area percentages.

#### 2.2.2. Quantification of Fatty Acid Contained in the Amaranth Oil by Gas Chromatography (GC)

Fatty acid transesterification was carried out as described by Eller and King [36]. Amaranth oil (1 mL) was mixed with 1 mL of a 10 mg/mL solution of triundecanoin in toluene along with 2 mL of 7% BF_3_ in methanol. The vial was sealed with a Teflon-lined screwcap and heated to 100 °C for 45 min with gentle mixing every 10 min. The vial was then allowed to cool to room temperature, and 5 mL of deionized water, 1 mL of hexane, and ca. 1 g of Na_2_SO_4_ were added and mixed vigorously. The vial was centrifuged to separate the layers, and the top layer was removed for subsequent GC-FAME analysis.

The fatty acid determination was performed following the methodology described by Eller et al. [37], and a Hewlett-Packard 5890 Series II GC (Palo Alto, CA, USA) with an SP 2380 column (30 m × 0.25 mm × 0.20 μm film thickness; Supelco, Bellefonte, PA, USA) was used. The parameters were: flow rate: 3.3 mL/min, helium head pressure: 0.14 MPa, split ratio: 22:1, programmed temperature ramp: 150–180 °C at 7 °C/min, 180–265 °C at 15 °C/min; injector and detector temperatures: 250 °C.

#### 2.2.3. Extraction and Characterization of Amaranth Unsaponifiable Matter by SFC

The extraction of unsaponifiable matter of the amaranth oil was conducted according to the AOCS Ca 6a-40 methodology [38]. Briefly, 30 mL 95% ethanol and 5 mL 5% KOH were added to 5 g of oil and boiled for 1 h in a reflux system. Once the solution reached room temperature, six washes with 50 mL petroleum ether were applied, followed by 25 mL 10% ethanol washes until the solution did not react with the phenolphthalein indicator. Calculations for the unsaponifiable matter concentration followed what was suggested by the protocol. The squalene concentration in the unsaponifiable matter was quantified by SFC following the procedure described in Section 2.2.1. Squalane (0.1 mg) was used as an internal standard. 

#### 2.2.4. Soybean Lunasin Purification by Anion Exchange Chromatography

Lunasin extract (LE) was obtained as reported previously, with slight modifications [31]. Briefly, freeze-dried lunasin-enriched soy extract, previously obtained [31] (40% *w/v*), was solubilized in 50 mL distilled water, centrifuged twice at 12,000× *g* for 10 min, and filtered through a 0.45 µm filter. A Hi-Trap Q HP column was used coupled with a Hi-Prep 26/10 desalting precolumn (GE Healthcare Bio-Sciences, Uppsala, Sweden). Unbound proteins were eluted with Tris-HCl (20 mM, pH 7.4) at a flow rate of 1 mL/min. Bound proteins were eluted using 0.4 M NaCl. A 280 nm wavelength UV lamp was used for lunasin detection. The LE sample was desalted using ultrafiltration through a 1 kDa disc, freeze-dried, and stored at −20 °C before being used in a liposome preparation for use in the in vitro analysis.

#### 2.2.5. Preparation of Liposomes Loaded with the Unsaponifiable Matter for Optimization and with Lunasin after Optimization

Optimization of the liposome formulation was carried out by a 3-factor, 3-level Box–Behnken design. The independent variables were DOPC:DOPA 1:1 M ratio, cholesterol, and amaranth unsaponifiable matter. The responses were particle size and encapsulation efficiency. Liposomes were prepared by the well-known methodology of thin-film hydration [21]. Three different concentrations of DOPC–DOPA, cholesterol, and amaranth unsaponifiable matter were dissolved in 500 µL of chloroform:methanol 2:1 *v/v*, followed by vortexing and drying with argon flow. Once a film formed at the bottom of the tubes, 1 mL of PBS was added for film rehydration. 

Particle size reduction was achieved by sonication with a probe sonicator model XL2000 (Misonix Incorporated, Farmingdale, NY, USA) for 15 s using an ice bath, and then the solution was filtered 10 times through a 0.2 µm syringe filter with an aluminum-based inorganic Anopore membrane (Cytiva, Marlborough, MA, USA). The liposome formulation containing lunasin was prepared using the optimized liposome with amaranth unsaponifiable matter for the hydrophobic phase. Lyophilized LE (49% LE/lipid ratio) was added after solvent (chloroform:methanol 2:1 *v/v*) evaporation. The lyophilized LE and lipids were solubilized in 500 μL of PBS pH 7.4 and left overnight (14 h) with continuous oscillatory shaking. Before decreasing the particle size as previously described, PBS was added to achieve a final volume of 1 mL.

To purify the liposomes from the free LE, the liposome solution was diluted with PBS (1:1 *v/v*) and centrifuged in Amicon filters at 12,000× *g* for 40 min to achieve a final volume of 1 mL in the retentate. 

#### 2.2.6. Liposome Characterization

##### Particle Size, Polydispersity Index (PDI), and Zeta Potential

The characterization of the liposomes was performed using a Malvern Zetasizer Nano Zs (Malvern, UK) at the Materials Research Laboratory, University of Illinois, USA. Ten microliters of the liposomal solution was diluted to a final volume of 1 mL with phosphate-buffered saline (pH 7.4). Diluted liposomes were transferred into a Folded Capillary Zeta Cell to be read by the method of dynamic light scattering (DLS) [39].

##### Encapsulation Efficiency (EE)

The liposomes were separated from the supernatant through centrifugation under the following conditions: 10,500× *g* for 20 min at 4 °C [40]; the supernatant was decanted, and the bilayer was disrupted with ethanol, followed by sonication in a water sonicator for 10 min. The squalene was quantified by SFC as indicated in Section 2.2.3. Squalane (0.1 mg) was used as the internal standard.

For lunasin encapsulation efficiency, LE protein in the filtrate obtained after liposome purification was quantified using DC protein assay for protein quantification as established by the manufacturer.

For EE, Equation (1) was used:

(1) $$EE=\left(\frac{m_{1}-m_{2}}{m_{1}}\right)\cdot 100$$

where EE = encapsulation efficiency, $m_{1}$ = total amount of encapsulated squalene (µg), and $m_{2}$ total amount of free squalene in the solution (µg).

The encapsulation efficiency was calculated based on the squalene concentration in the unsaponifiable matter.

##### Transmission Electron Microscopy (TEM)

Holey carbon-coated copper grids were glow discharged for 25 s with a PELCO easiGlow Glow Discharge System (Ted Pella, Inc., Redding, CA, USA). Vitrified liposomes were prepared with a ThermoScientific Vitrobot Mark IV System (FEI, Hillsboro, OR, USA). Vitrobot was set to 4 °C and 100% relative humidity; a 3 μL aliquot was applied to the grid, blotted for 3 s, and plunged into liquid ethane. Liposomes were imaged using a ThermoFisher Glacios Cryogenic Transmission Electron Microscope operating at 200 kV. Images were collected using a ThermoFisher Ceta-D CMOS camera

TEM-imaged blank liposome formulation was analyzed with ImageJ software (NIH, Bethesda, MD, USA) [41]. After setting the scale, the Gaussian blur filter and brightness/contrast were modified to improve the particle edge visualization. The particle size was measured by manually measuring the diameter of the particles, with at least four repetitions per particle.

#### 2.2.7. Determination of Cell Cytotoxicity after Treatments with Empty Liposomes, Loaded with the Unsaponifiable Matter and Loaded with Lunasin

In vitro cytotoxicity tests were conducted to determine whether the liposome formulation was effective against melanoma, B16-F10 mouse melanoma (ATCC CRL6475), and A-375 [A375] (ATCC CRL-1619) human skin melanoma (American Type Culture Collection, Manassas, VA, USA). Cells were inoculated in 96-well plates at a confluence of 1 × 10^5^ cells per well. Since the dilution of amaranth unsaponifiable matter with a safe concentration of dimethyl sulfoxide (DMSO) for cells was not achieved, different concentrations of pure squalene solubilized in <0.05% DMSO (from 10 nM to 2 mM) were tested in cell lines A-375 and B16-F10 to determine the cytotoxicity of the triterpene and, thus, the potential cytotoxicity of the amaranth unsaponifiable matter. In addition, blank liposomes, liposomes loaded with amaranth unsaponifiable matter, liposomes with lunasin (not UM = unsaponifiable matter), and liposomes with the unsaponifiable matter and lunasin from 0.42 to 1.68 mg/mL were tested. All treatments were performed within 24 h. Cells were maintained at 37 °C, 5% CO_2_, and 95% air. Cell viability was measured using CCK-8 according to the manufacturer’s protocol (Abcam ab228554). 

#### 2.2.8. Statistical Analysis

Liposome optimization was performed using a Box–Behnken surface response methodology using Design Expert 12 software (Stat-Ease Inc., Minneapolis, MN, USA). The optimal conditions were predicted by a second-order polynomial model. The general regression equation used was:

(2) $$Y=\beta_{0}+\beta_{1}X_{1}+\beta_{2}X_{2}+\beta_{3}X_{3}+\beta_{12}X_{1}X_{2}+\beta_{13}X_{1}X_{3}+\beta_{23}X_{2}X_{3}+\beta_{11}X_{1}^{2}+\beta_{22}X_{2}^{2}+\beta_{33}X_{3}^{2}$$

where Y is the predicted response; *β*_0_ is the model constant; *X*_1_, *X*_2_, and *X*_3_ are independent variables; *β*_1_, *β*_2_, and *β*_3_ are quadratic coefficients; *β*_12_, *β*_13_, and *β*_23_ are cross-product coefficients; and *β*_11_, *β*_22_, and *β*_33_ are quadratic coefficients. 

GraphPad Prism 8 (GraphPad Software Inc., San Diego, CA, USA) was used for the cell viability analysis. Experimental data were tested for normality and analyzed by one-way analysis of variance with a Tukey post hoc test. A *p*-value < 0.5 was considered statistically significant.

## 3. Results and Discussion

**Figure 1:**
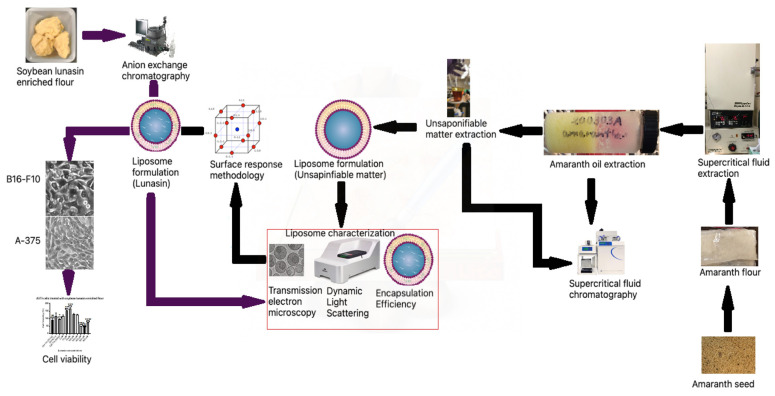
Experimental diagram.

The aims of this study were to load lunasin into optimized liposomes as a potential antimelanoma bioactive peptide, testing liposomes in human and mouse melanoma cells, and to improve the lunasin dose response to inhibit 50% of the cells. Figure 1 presents the experimental diagram.

### 3.1. Amaranth Oil Extracted by Supercritical Fluid Extraction and Squalene Determination using Supercritical Fluid Chromatography

**Figure 2:**
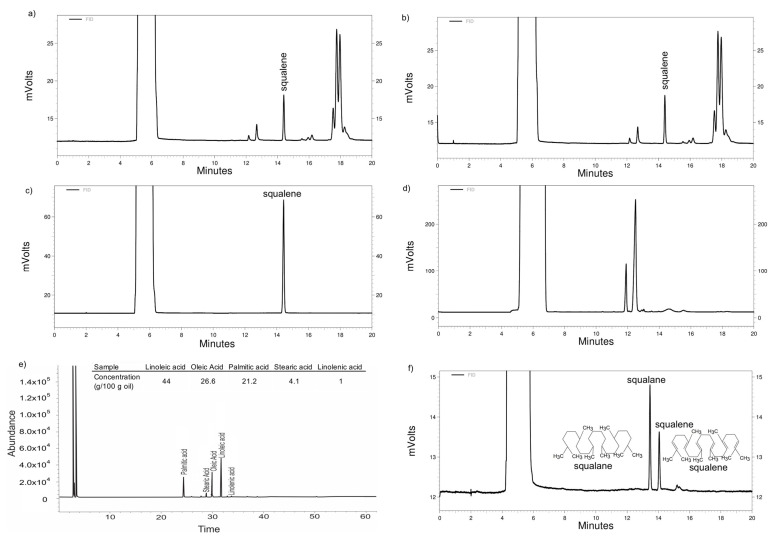
Chromatograms of the squalene content in (**a**) supercritical fluid chromatogram (SFC) of the amaranth oil extracted using supercritical fluid extraction (SFE) at 27.6 MPa, 40 °C, solvent (CO_2_)-to-feed (Oil) ratio 20. (**b**) SFC of the amaranth oil extracted using SFE at 55.2 MPa, 80 °C, solvent (CO_2_)-to-feed (Oil) ratio of 20. (**c**) SFC of high-purity squalene standard. (**d**) SFC showed that squalene was degraded after transesterification with BF_3_. (**e**) Gas chromatogram after lipid transesterification and fatty acid concentration in the amaranth oil. (**f**) SFC of squalene content in the optimized liposome used for encapsulation efficiency calculation; squalane was added as an internal standard.

The extraction conditions that led to the highest oil yield were: pressure of 55.2 MPa, temperature of 80 °C, and solvent (CO_2_) to feed (oil) ratio of 20. The oil yield was 7.19 ± 0.01 g per 100 g of seed with a squalene concentration of 36.64 ± 0.64 g per 100 g of oil before unsaponifiable matter extraction (Figure 2). The extraction yield on this investigation was similar to that obtained by Krujl et al. [2]. These investigators reported oil yields ranging from 5.88% to 6.61% when using SFE at 40 °C/20.68 MPa in different *Amaranthus* sp. genotypes. 

Regarding squalene in amaranth oil, Rosales et al. [4] achieved a 46.00 ± 2.81% squalene yield in the oil fraction extracted by SFE at 40 °C/20.68 MPa. Kraujalis and Venskutonis [42] extracted oil from amaranth seeds through SFE, adding 0%, 2%, or 5% ethanol as a cosolvent with six different pressure conditions. The highest yield of oil at 0%, 2%, and 5% ethanol was 4.37 ± 0.41, 4.74 ± 0.21, and 5.12 ± 0.24 g/100 g seed, respectively. It was shown that 5% ethanol as a cosolvent extracted a higher amount of oil, which increased with increased pressure, obtaining 3.20 ± 0.09 g/100 g seed at 15 MPa and 5.12 ± 0.24 g/100 g seed at 0.55 MPa.

### 3.2. Fatty Acid Transesterification and Extraction of Unsaponifiable Matter from Amaranth Oil

As expected, no squalene was seen in amaranth oil after transesterification with BF_3_ (Figure 2), so the material was only used for fatty acid detection. 

The main fatty acids present in amaranth oil were unsaturated: linoleic and oleic acids (44 and 26.6 g/100 g oil, respectively) (Figure 2). Shukla et al. [43] reported similar concentrations for both fatty acids: 47.34 and 27.48 g/100 g oil, respectively. 

The unsaponifiable matter in most vegetables and seeds is composed mostly of sterols [44]. However, the squalene in amaranth oil could be the most important constituent of the unsaponifiable matter and can accumulate up to 5% (*w/w*) [42,45]. In the present research, amaranth oil contained 4.26 g of unsaponifiable matter per 100 g of oil. After the extraction of unsaponifiable matter from the amaranth oil, the squalene concentration was 25.49 ± 0.02 g of squalene per 100 g of unsaponifiable matter, which represented 75.82 ± 3.18 mg of squalene per 100 g seed. Dhellot et al. [46], using a different lipid extraction method in two varieties of *A. hybridus*, extracted unsaponifiable matter in the range from 5.27 ± 0.57% to 7.21 ± 0.47%. In a different study, 104 genotypes of 30 species of amaranth were analyzed with regard to oil and squalene content. The squalene concentration in these species ranged from 13 to 560 mg per 100 g seed, with the average concentration being 213 mg squalene per 100 g seed [47]. Differences in the squalane and squalene chemical structures can be seen in Figure 2.

### 3.3. Liposome Optimization with Amaranth Unsaponifiable Matter Using Response Surface Methodology (Box–Behnken Design)

The characterization of the liposomes (Table 1) showed a particle size that ranged from 115.80 ± 8.77 to 163.12 ± 15.68 nm with a squalene encapsulation efficiency from 33.14 ± 13.18 to 76.08 ± 14.75%. Figure 2 presents the SFC of squalene content on the optimized liposome used for the squalene EE calculation.

The suggested model was a two-factor interaction for particle size response and quadratic for squalene EE (Table 2). 

The *p*-value for the particle size mathematical model was 0.01, *R*^2^ = 0.83, and a lack of fit *p* > 0.05, meaning that the model is reliable. The fitted equation was:

(3) $$Y=132.23-1.43X_{1}+9.68X_{2}+5.60X_{3}+1.17X_{1}X_{2}-1.34X_{1}X_{3}+12.38X_{2}X_{3}$$

The *p*-value for the squalene EE mathematical model was 0.02, *R*^2^ = 0.93, and a lack of fit *p* > 0.05, also indicating that the model is reliable. The fitted equation was:

(4) $$Y=74.26-8.32X_{1}+0.0175X_{2}-2.19X_{3}-1.44X_{1}X_{2}+1.34X_{1}X_{3}-10.14X_{2}X_{3}-4.23X_{1}^{2}-13.02X_{2}^{2}+13.88X_{3}^{2}$$

**Figure 3:**
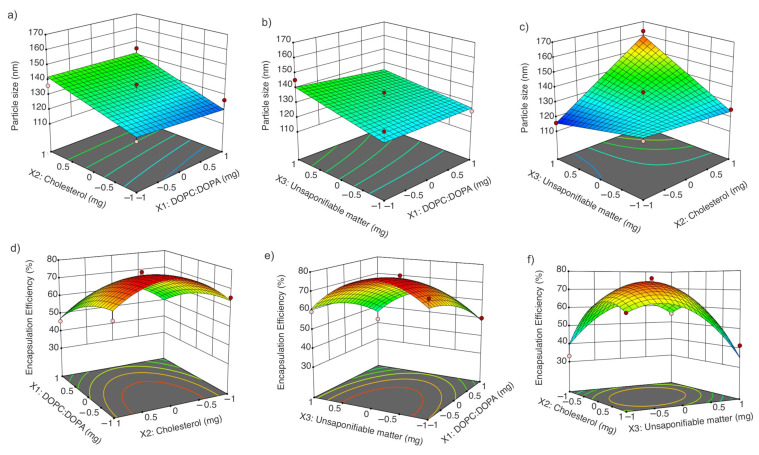
Response surfaces and contours: (**a**) X_2_ and X_1_ interaction in particle size response; (**b**) X_3_ and X_1_ interaction in particle size response; (**c**) X_3_ and X_2_ interaction in particle size response; (**d**) X_1_ and X_2_ interaction in encapsulation efficiency response; (**e**) X_3_ and X_1_ interaction in encapsulation efficiency response; (**f**) X_2_ and X_3_ interaction in encapsulation efficiency response.

The interactions of the factors are shown in Figure 3. A significant interaction was found between the unsaponifiable matter (X_3_) and cholesterol (X_2_) for particle size. For squalene EE, significance was seen in terms of the 1,2-dioleoyl-sn-glycero-3-phosphocholine:1,2-dioleoyl-sn-glycero-3-phospho-rac-(1-glycerol) sodium salt (DOPC:DOPA) concentration (X_1_), unsaponifiable matter (X_3_), and cholesterol (X_2_) interaction, as well as the quadratic effects of cholesterol (X_2_) and unsaponifiable matter (X_3_) (Table 2). 

#### 3.3.1. Effect of DOPC:DOPA Ratio, Cholesterol, and Amaranth Unsaponifiable Matter on Particle Size

The type of hydrocarbon chain attached to the glycerol affected the particle size. Monounsaturated fatty acids, such as oleic, could develop larger liposomes compared to saturated fatty acids due to the packing parameter; unsaturated fatty acids produce imperfect packing in the membrane [48]. However, the use of phospholipids with unsaturated fatty acids with a low transition temperature can lead to higher flexibility of the liposomes, which is the reason for selecting DOPC over other phospholipids such as POPC [49,50]. 

**Figure 4:**
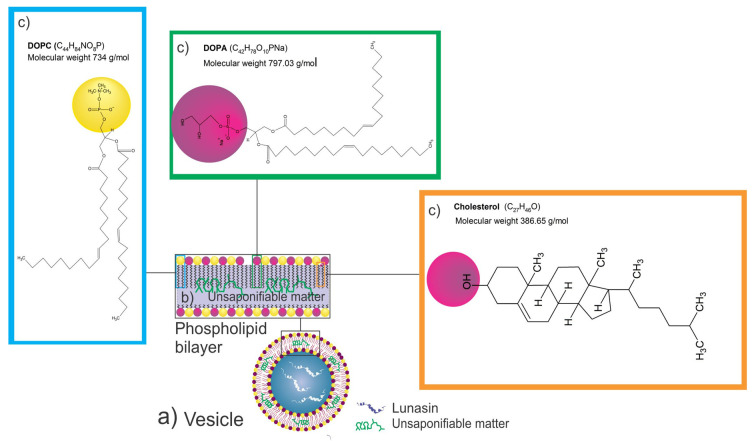
Expected liposome conformation diagram. (**a**) Vesicle (liposomes) loaded with lunasin (white structures) and amaranth unsaponifiable matter (green structures); (**b**) expected amaranth unsaponifiable matter location; (**c**) chemical structures and location of phospholipids (DOPC:DOPA) and cholesterol.

Due to the transition temperatures of DOPC and DOPA, −17 and −4 °C, respectively, the formulation was fluid at room temperature, increasing the flexibility of the particle (not related to the solubility of the preparation). Phosphatidylcholine is the most commonly occurring zwitterionic lipid that provides a net charge equal to 0 [51]. Due to its net charge, a vesicle prepared only with DOPC will aggregate in the formulation; the incorporation of DOPA, which has a net charge equal to −1, will allow the liposomes to be dispersed in the formulation (Figure 4). The unsaponifiable matter of amaranth was expected to be located between the liposome bilayer (Figure 4). 

The interaction between cholesterol and amaranth unsaponifiable matter affected the particle size; the higher the concentration of cholesterol/ amaranth unsaponifiable matter, the larger the particle size. The opposite behavior was observed for the DOPC:DOPA ratio: the higher the DOPC:DOPA ratio, the smaller the particle size (Figure 3a–c). Cholesterol affects the vesicle rigidity, thickness, stability, fluidity, and size, as shown by Kaddah et al. [52]. The addition of cholesterol to the liposomes increased the particle size; for instance, the addition of 30% cholesterol with respect to the phospholipid concentration increased the particle size by more than 2-fold compared with the particles with no cholesterol added; the reported particle sizes were 472.00 ± 0.54 nm and 220.00 ± 3.72 nm, respectively.

**Figure 5:**
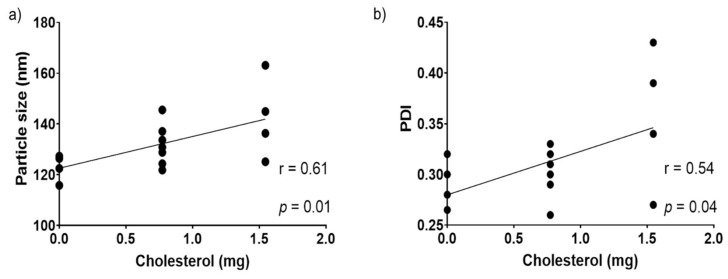
(**a**) Correlation between cholesterol and particle size; (**b**) correlation between cholesterol and PDI.

Correlations of all parameters (Table 3, Figure 5) suggested that particle size and PDI were positively affected by the cholesterol concentration; a higher cholesterol concentration produced a higher particle size and PDI. In a study, liposomes were prepared with 300, 600, and 900 mg Phosal and 0, 50, 100, and 150 mg cholesterol in each Phosal formulation. Particle size increased significantly when 150 mg of cholesterol was added in all formulations [53]. 

#### 3.3.2. Effect of DOPC:DOPA Ratio, Cholesterol, and Amaranth Unsaponifiable Matter on Encapsulation Efficiency

The DOPC:DOPA ratio, as well as the cholesterol and unsaponifiable matter interaction, significantly affected the squalene EE (Table 2 and Figure 3d–f). The SRM suggested that a concentration close to 0 for both cholesterol and unsaponifiable matter and a ratio close to −1 for DOPA:DOPC increased the squalene EE. Hudiyanti et al. [54] tested the encapsulation of beta-carotene in liposomes prepared with different cholesterol concentrations. It was shown that 0% cholesterol produced an encapsulation efficiency of 71.34%, while 40% cholesterol decreased the encapsulation efficiency to 48.04%. These results suggest a competition between cholesterol and beta-carotene molecules in the lipid bilayer. Figure 4 presents a diagram of the expected liposome conformation, including lunasin. In contrast, when vitamin C was added to the vesicles instead of beta-carotene, the encapsulation efficiency increased from 57.13% to 85.16% with 0% and 40% cholesterol, respectively, due to the lower lipid bilayer permeability. Cholesterol improved the hydrophilic drug retention by bilayer stabilization through the hydration of the polar groups on the surfaces [55].

Tocopherols are molecules that could improve the stability of the liposome bilayer when cholesterol is absent (Figure 4). Tocopherols play a role similar to cholesterol. When tocopherol is present in the liposome bilayer, it decreases the normal leakage of the aqueous core [56]. Since amaranth unsaponifiable matter contains alpha-, beta-, gamma-, and delta-tocopherol [42], further research is needed to determine whether tocopherols play a similar role to cholesterol, preventing the leakage of hydrophilic molecules.

#### 3.3.3. Optimization of Liposomes Loaded with the Unsaponifiable Matter

The optimization of the liposome was aimed to increase the phospholipid concentration to enhance the encapsulation of hydrophilic molecules. The suggested conditions to maximize the phospholipid concentration and squalene EE and minimize the particle size were: X_1_ (DOPA:DOPC) 15.27 mg, X_2_ (cholesterol) 0 mg, and X_3_ (amaranth unsaponifiable matter) 1.1 mg, with a predicted particle size of 117.09 ± 6.60 nm and a squalene EE of 54.25 ± 6.04%. The optimized liposome did not contain cholesterol. Hudiyanti et al. [57] showed that the encapsulation efficiency of β-carotene decreased from 79% to 60% with 0% and 40% cholesterol, respectively, since both molecules competed to be inside the vesicle bilayer. Quinine entrapment by liposomes was also affected by the cholesterol incorporation in the system, where 40% cholesterol produced an EE ranging from 5.6% to 84.07%; decreasing the cholesterol by 10% increased the EE to 88% [58]. Three independent samples of optimized liposomes were tested; the results of the optimized liposomes were 121.3 ± 4.81 nm particle size and 58.97 ± 0.12% squalene EE, as predicted, and −79.21 ± 11.18 mV zeta potential. Once the liposome was optimized, lunasin was added to challenge the liposomes against melanoma cancer cell lines B16-F10 and A-345. The characterization of these liposomes showed 82.14 ± 3.34% lunasin EE, 128.60 ± 1.28 nm particle size, 0.28 ± 0.01 PDI, and −75.91 ± 6.63 mV zeta potential. A similar EE was reported in liposomes loaded with anionic and cationic whey peptides, which showed peptide EE values of 88.30% and 92.90%, respectively [59].

Hydrophilic molecules’ encapsulation is enhanced by the phospholipid concentration. Encapsulation of acetylcholinesterase, an enzyme loaded into the liposome aqueous phase, showed a linear relationship between the EE and phospholipid concentration. While 0.5 mg/mL of phospholipids resulted in ~3% EE, 2 mg/mL of phospholipids showed ~40% EE [60]. Briuglia et al. [58] tested three different phospholipids in combination with cholesterol in ratios of 70:30%, 60:40%, and 50:50% (phospholipid:cholesterol). It was shown in this study that EE reached levels higher than 90% in a ratio of 70:30, 88% in a ratio of 60:40, and 87% in a ratio of 50:50.

An important parameter to measure while preparing particles is the zeta potential since it determines the electrostatic repulsion between particles; the stability could be affected by the particle net charge. The probability of aggregation increases when the zeta potential gets close to 0 mV [21,61,62]. The zeta potential of the different liposomal solutions was between –76.62 ± 2.00 mV and –95.30 ± 10.47 mV, which means the preparations were considered stable.

### 3.4. Transmission Electron Microscopy (TEM) Images of Empty Liposomes, Optimized Liposomes, and Lunasin-Loaded Liposomes

**Figure 6:**
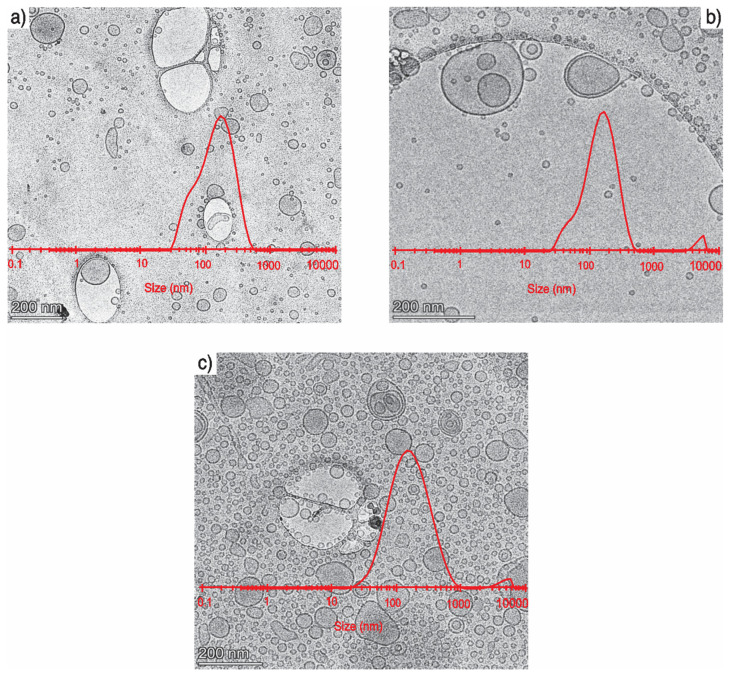
Transmission electron micrographs of liposomes. (**a**) The blank liposome contained DOPC:DOPA in a 1:1 M ratio. (**b**) The optimized liposome contained DOPC–DOPA (15.27 mg/mL) and amaranth unsaponifiable matter (1.1 mg/mL). (**c**) The optimized liposome with lunasin containing DOPA–DOPC (15.27 mg/mL), amaranth unsaponifiable matter (1.1 mg/mL), and lunasin (8 mg/mL). All samples were prepared with a 1 mg/mL lipid concentration. Bars are equivalent to 200 nm. The graph represents the particle size distribution, determined by dynamic light scattering. The larger structures are part of the holey carbon film, which contains unobstructed regions to improve the sample image by reducing the noise from the background. A picture of the holey carbon coat can be found at the website of the supplier: https://www.2spi.com/category/grids-custom-holey-carbon/, accessed 14 July 2021.

Vitrified liposomes imaged by Cryo-TEM are shown in Figure 6. To obtain the particle size distribution, the mean particle size was manually measured as 50.43 ± 26.70 nm, 48.12 ± 32.84 nm, and 46.43 ± 25.13 nm for blank liposomes, optimized liposomes, and lunasin-loaded liposomes, respectively; however, DLS showed a particle size of 116.48 ± 1.31 nm, 121.33 ± 4.81 nm, and 129.0 ± 1.34 nm. The difference in particle size is due to differences in the analytical methods used. DLS measures the hydrodynamic size of the particles by light scattering from the laser beam that passes through the solution [63]. Hydrodynamic size is usually larger than physical diameter due to the inclusion of hydration; the size is also influenced by the interactions of the particle with the medium [64]. The particles could be surrounded by different molecules that are not ingredients of the particle itself; these molecules scatter the light, so that in DSL, the particles analyzed are affected by the surrounded molecules, which changes the composition and surface chemistry [65]. Another reason why DLS tends to overestimate the larger particles is that the scattered light intensity strongly depends on the size of scattering objects. Meanwhile, TEM measures the dry size of the nanoparticles; it does not include the water or the interaction of the particles with the medium [64].

### 3.5. Melanoma B16-F10 and A-375 Cell Viability Treated with Empty Liposomes, Liposomes Loaded with Unsaponifiable Matter (Optimized), and Lunasin-Loaded Liposomes

**Figure 7:**
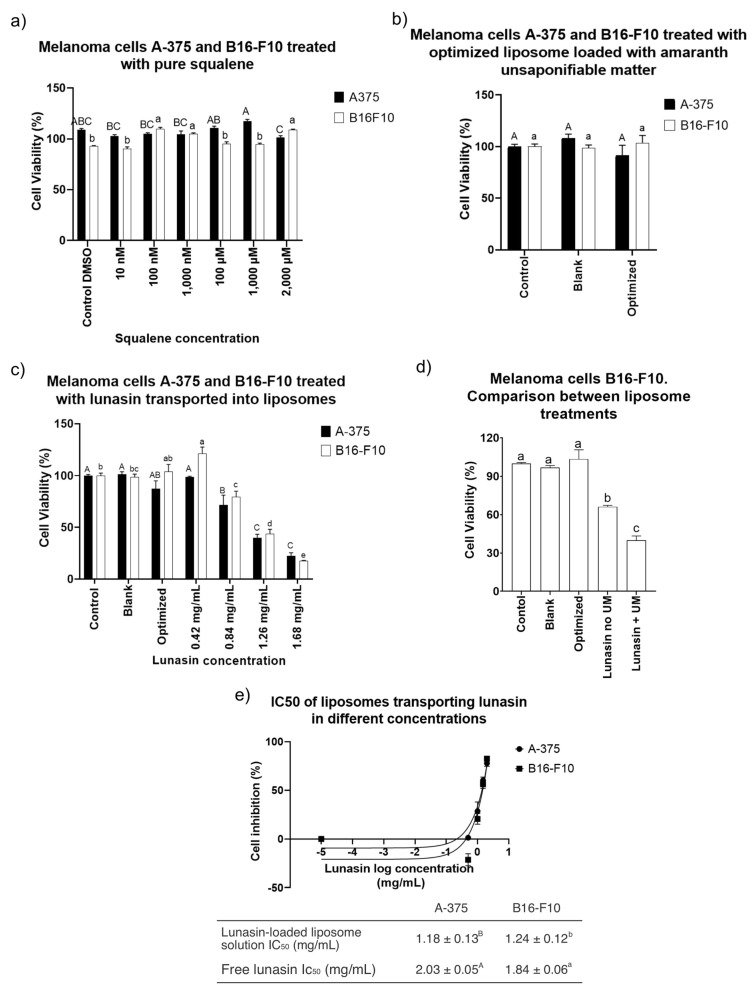
Cell viability assay performed with CCK-8 (2-(2-methoxy-4-nitrophenyl)-3-(4-nitrophenyl)-5-(2,4-disulfophenyl)-2H-tetrazolium, monosodium salt) in both cell lines. (**a**) A-375 and B16-F10 cells treated with different concentrations of pure squalene dissolved in DMSO (<0.05%). (**b**) A-375 and B16-F10 cells treated with blank liposomes prepared with DOPC–DOPA (15.27 mg/mL) and optimized liposomes prepared with DOPC–DOPA loaded with amaranth unsaponifiable matter. The control was treated with PBS. (**c**) A-375 and B16-F10 cells treated with blank liposomes prepared with DOPC–DOPA; optimized liposome prepared with DOPC–DOPA loaded with amaranth unsaponifiable matter; optimized liposome prepared with DOPC–DOPA, amaranth unsaponifiable matter, and different concentrations of soybean lunasin extract (0.42 to 1.68 mg/mL). The control was treated with PBS. (**d**) B16-F10 cells treated with blank liposomes prepared with DOPC–DOPA; optimized liposomes prepared with DOPC–DOPA loaded with amaranth unsaponifiable matter; liposome prepared with DOPC–DOPA and lunasin 1.26 mg/mL (no unsaponifiable matter); optimized liposome prepared with DOPC–DOPA, amaranth unsaponifiable matter, and soybean lunasin extract (1.26 mg/mL). The control was treated with PBS. (**e**) IC_50_ of optimized liposomes loaded with soybean lunasin extract prepared the same way as liposomes in (**c**). A comparison between cells treated with lunasin-loaded liposomes and cells treated with free lunasin (nonencapsulated lunasin). DOPC–DOPA = 15.27 mg/mL; amaranth unsaponifiable matter = 1.1 mg/mL; capital letters indicate significant differences in A-375 cells (*p* < 0.001); lowercase indicates a significant difference in B16-F10 cells (*p* < 0.0001). Abbreviations: UM: unsaponifiable matter; Control DMSO: control treated with dimethyl sulfoxide (<0.05%); IC_50_: half-maximum inhibitory concentration.

There was a significant increase in B16-F10 cell viability after treatment with pure squalene (Figure 7a). B16-F10 cell viability increased up to 109.67 ± 0.03% (*p* < 0.0001) with respect to a control-treated with DMSO. A549 adenocarcinoma cells treated with up to 100 μM did not have altered cell viability after 14 days of incubation [66]. Warleta et al. [67] measured proliferation in MCF7, MDA-MB-231, and MCF 10A breast cancer cells after 24, 48, and 120 h of treatment with squalene up to 50 μM. No influence of squalene on cell survival was reported; instead, they found a nonsignificant increase in MDA-MB-231 cells. 

Similar behavior was observed in the present study after treatment of both cell lines with liposomes carrying amaranth unsaponifiable mater (1.1 mg/mL) (Figure 7b), where there was a nonsignificant effect after 24 h treatment (Figure 7b). However, after the incorporation of lunasin into the optimized liposome (Figure 7c), significant dose-dependent behavior was shown in both cell lines (Figure 7c). Free lunasin showed a half-maximum inhibitory concentration (IC_50_) after 24 h equal to 1.84 ± 0.06 mg/mL (equivalent to 330 μM) in B16-F10 and IC_50_ = 2.03 ± 0.05 mg/mL (equivalent to 370 μM) in A-375. In a study where melanoma A-375 cell line was used, 100 μM free lunasin (equivalent to 0.5 mg/mL) was unable to decrease the cell viability below ± 80% [30].

The purpose of loading unsaponifiable matter as a source of SQ into the liposomes was to potentiate the anticancer activity of liposomes with lunasin. SQ reduced the effects of chemical carcinogenic promoters when applied to mouse skin tumors. In addition, SQ has protective effects against the carcinogen 12-O-tetradecanoylphorbol-13-acetate in mouse skin [18]. After the treatment of melanoma cell line B16-F10 using liposomes prepared with DOPC–DOPA (15.27 mg/mL) plus lunasin (1.24 mg/mL) and no unsaponifiable matter, the cell viability was 66.04 ± 0.02% (*p* < 0.0001). Compared with the treatment of liposomes with DOPC–DOPA (15.27 mg/mL) encapsulating amaranth unsaponifiable matter and lunasin, there was a significant difference (*p* < 0.0001) between the treatments (Figure 7d).

Lunasin is a 5 kDa peptide. Based on electrophoresis and Western blot, the lunasin purity was high (Figure S1). As we observed on the high-molecular-weight gel, the proteins obtained after purification were below 10 kDa. After applying the purified lunasin to low-molecular-weight gels, the bands were located around the 5 kDa region, and there was a positive response of those bands to the lunasin antibody.

The incorporation of lunasin into liposomes improved cancer cell cytotoxicity, decreasing IC_50_ after 24 h treatment in both cell lines to 1.24 ± 0.12 mg/mL (equivalent to 225 μM) in B16-F10 and to 1.18 ± 0.13 mg/mL (equivalent to 215 μM) in A-375 (Figure 7e).

The results of this study agree with previous preparations of liposomes with cyclic peptides. A similar decrease in the IC_50_ was found by Kilian et al. [68] when MCF-7 cells were treated with the dipeptides cyclo(His-Ala) and cyclo(His-Gly) in two different forms, free and encapsulated into liposomes. The reported IC_50_ for MCF-7 cells treated with cyclo(His-Ala) was 1.258 mM when it was administered freely and 0.498 mM when it was loaded into liposomes; the IC_50_ for the same cells treated with the dipeptide cyclo(His-Gly) decreased from 0.630 to 0.358 mM.

## 4. Conclusions

The SFE extraction conditions affected the squalene extraction efficiency. In this study, 55.2 MPa pressure and 80 °C were the best conditions for squalene extraction. 

The optimal condition for liposomes loaded with amaranth unsaponifiable matter did not contain cholesterol but included the highest amount of DOPC–DOPA. Optimization in this study aimed to minimize particle size and to maximize squalene encapsulation efficiency. For future studies, it is recommended to characterize the unsaponifiable matter by the different sterol and tocopherol profiles to learn about their effect on encapsulation efficiency.

Cell treatment with squalene incorporated into liposomes seems to play a protective role in melanoma cells. The cancer cell cytotoxicity of lunasin in melanoma cells improved by its incorporation into liposomes decreasing the IC_50_ by 31.81% in B16-F10 and 41.89% in A-375, compared with free lunasin treatments.

## Figures and Tables

**Table 1:** Observed and predicted values from the Box–Behnken Design analysis and characterization of the different liposome formulations for the optimization.

#	Factors			Size (nm)		Squalene EE (%)		Zeta Potential (mV)	PDI
	DOPC:DOPA X_1_ (mg)	Cholesterol X_2_ (mg)	Amaranth Unsaponifiable Matter X_3_ (mg)	Observed	Predicted	Observed	Predicted		
1	−1 (9.50)	0 (0.77)	1 (1.11)	145.50 ± 2.17	140.61	59.45 ± 5.64	45.11	−86.43 ± 1.50	0.33 ± 0.03
2	−1 (9.50)	1 (1.55)	0 (0.89)	136.30 ± 2.87	142.17	62.01 ± 10.93	58.1	−76.62 ± 1.42	0.34 ± 0.03
3	0 (12.67)	−1 (0.00)	1 (1.11)	115.80 ± 8.77	115.77	55.16 ± 1.81	56.03	−84.18 ± 6.32	0.28 ± 0.02
4	1 (15.83)	−1 (0.00)	0 (0.89)	126.40 ± 3.67	119.94	54.87 ± 3.66	51.11	−81.92 ± 3.52	0.30 ± 0.00
5	0 (12.67)	0 (0.77)	0 (0.89)	128.80 ± 2.73	132.23	72.58 ± 7.10	50.77	−82.42 ± 2.52	0.31 ± 0.04
6	−1 (9.50)	−1 (0.00)	0 (0.89)	122.48 ± 1.65	125.16	65.47 ± 6.16	47.98	−82.22 ± 0.55	0.27 ± 0.00
7	0 (12.67)	0 (0.77)	0 (0.89)	121.82 ± 1.05	132.23	74.12 ± 0.81	50.77	−80.57 ± 1.70	0.26 ± 0.00
8	0 (12.67)	1 (1.55)	1 (1.11)	163.12 ± 15.68	159.90	41.29 ± 16.34	38.19	−95.30 ± 7.40	0.43 ± 0.02
9	0 (12.67)	−1 (0.00)	−1 (0.66)	127.32 ± 5.02	129.33	33.14 ± 13.18	43.06	−84.95 ± 1.95	0.32 ± 0.04
10	0 (12.67)	1 (1.55)	−1 (0.66)	125.10 ± 3.43	123.92	59.83 ± 13.58	65.78	−82.43 ± 6.03	0.27 ± 0.01
11	1 (15.83)	1 (1.55)	0 (0.89)	144.92 ± 11.95	141.65	45.65 ± 0.00	45.87	−92.92 ± 3.15	0.39 ± 0.08
12	−1 (9.50)	0 (0.77)	−1 (0.66)	133.65 ± 3.48	126.72	72.63 ± 0.37	60.97	−80.90 ± 1.03	0.30 ± 0.03
13	1 (15.83)	0 (0.77)	1 (1.11)	130.82 ± 0.05	135.06	42.34 ± 9.27	49.11	−80.25 ± 5.25	0.29 ± 0.02
14	0 (12.67)	0 (0.77)	0 (0.89)	137.10 ± 15.73	132.23	76.08 ± 14.75	50.77	−82.67 ± 3.77	0.32 ± 0.07
15	1 (15.83)	0 (0.77)	−1 (0.66)	124.33 ± 3.07	126.53	50.16 ± 8.86	47.87	−82.77 ± 0.30	0.29 ± 0.02

Results are expressed as the mean ± standard error of two independent replicates. Abbreviations: #: experiment number EE: encapsulation efficiency; PDI: polydispersity index.

**Table 2:** Surface response model ANOVA table per output variable (fitted model).

	Encapsulation Efficiency (Quadratic)				Particle Size (Two Factors Interaction)			
	Sum of Squares	df	Mean Square	*p*-Value	Sum of Squares	df	Mean Square	*p*-Value
**Model**	2280.95	9.00	253.44	**0.02**	1643.29	6.00	273.88	**0.01**
X_1_ (DOPC:DOPA)	553.45	1.00	553.45	**0.01**	16.44	1.00	16.44	0.56
X_2_ (Cholesterol)	0.00	1.00	0.00	0.99	749.49	1.00	749.49	**<0.01**
X_3_ (Unsaponifiable matter)	38.37	1.00	38.37	0.35	251.25	1.00	251.25	**0.04**
X_1_X_2_	8.29	1.00	8.29	0.65	5.52	1.00	5.52	0.73
X_1_X_3_	7.18	1.00	7.18	0.68	7.20	1.00	7.20	0.69
X_2_X_3_	411.28	1.00	411.28	**0.02**	613.39	1.00	613.39	**0.01**
X_1_^2^	66.22	1.00	66.22	0.24	-	-	-	-
X_2_^2^	626.40	1.00	626.40	**0.01**	-	-	-	-
X_3_^2^	711.34	1.00	711.34	**0.01**	-	-	-	-
**Residual**	182.25	5.00	36.45	-	348.51	8.00	43.56	-
Lack of Fit	176.10	3.00	58.70	>0.05	231.43	6.00	38.57	0.71
Pure Error	6.15	2.00	3.08	-	117.08	2.00	58.54	-

**Table 3:** Correlation coefficients between variables.

Correlation Matrix	DOPC:DOPA Ratio	Cholesterol	Unsaponifiable Matter	Particle Size	Encapsulation Efficiency	PDI	Zeta Potential
DOPC:DOPA ratio	1.00						
Cholesterol	0.00	1.00					
Unsaponifiable matter	0.00	0.00	1.00				
Particle size	−0.09	0.61 *	0.36	1.00			
Encapsulation efficiency	−0.47	0.86	−0.12	−0.25	1.00		
PDI	0.07	0.54 *	0.31	0.91	−0.43	1.00	
Zeta Potential	−0.23	−0.28	−0.30	−0.68	0.50	−0.76	1.00

* *p* < 0.05, significant correlations are in bold. DOPA:DOPC ratio, 1,2-dioleoyl-sn-glycero-3-phosphocholine:1,2-dioleoyl-sn-glycero-3-phospho-rac-(1-glycerol) sodium salt; PDI, polydispersity index.

## Data Availability

The study did not report any data.

## Supplementary Materials

The following are available online at https://www.mdpi.com/article/10.3390/nano11081960/s1, Figure S1: Lunasin purity after extraction. (a) High molecular weight gel electrophoresis. (b) Low molecular weight gel electrophoresis. (c) Western blot.
