# Supplementary material for: Liposomes Loaded with Unsaponifiable Matter from Amaranthus hypochondriacus as a Source of Squalene and Carrying Soybean Lunasin Inhibited Melanoma Cells
Source: Nanomaterials (Basel). 2021 Jul 30;11(8):1960. doi: 10.3390/nano11081960 (PMC8397957; doi:10.3390/nano11081960)
Supplement: Supplementary file 1 [file nanomaterials-11-01960-s001.zip › nanomaterials-1295091-supplementary.pdf]

nanomaterials

MDPI

Supplementary Materials

Liposomes Loaded with Unsaponifiable Matter from *Amaranthus hypochondriacus* as a Source of Squalene and Carrying Soybean Lunasin Inhibited Melanoma Cells

Erick Damian Castañeda-Reyes [1,2], Elvira Gonzalez de Mejia [1,*], Fred Joseph Eller [3], Mark A. Berhow [3], María de Jesús Perea-Flores [4] and Gloria Dávila-Ortíz [2,*]

[1] Department of Food Science and Human Nutrition, University of Illinois, Urbana-Champaign, IL, 61801, USA; edreyes@illinois.edu

[2] Departamento de Ingeniería Bioquímica, Escuela Nacional de Ciencias Biológicas, Instituto Politécnico Nacional (IPN), Av. Wilfrido Massieu, esq. Miguel Stampa s/n, Zacatenco, Alcaldía Gustavo A. Madero, Ciudad de México 07738, México

[3] Functional Foods Research, United States Department of Agriculture, National Center for Agricultural Utilization Research†, Midwest Area, Agricultural Research Service, 1815 N. Univ. St., Peoria, IL 61604, USA; fred.eller@usda.gov (F.J.E.); mark.berhow@usda.gov (M.A.B.)

[4] Centro de Nanociencias y Micro y Nanotecnologías, Instituto Politécnico Nacional (IPN), Av. Luis Enrique Erro s/n, Unidad Profesional Adolfo López Mateos, Zacatenco, Alcaldía Gustavo A. Madero, Ciudad de México 07738, México; mpereaf@ipn.mx

* Correspondence: edemejia@illinois.edu (E.G.d.M.); gdavilao@ipn.mx (G.D.-O.); Tel.: +1(217)-244-3196 (E.G.d.M.); +52-(55)-5729-6000 (ext. 57870) (G.D.-O.)

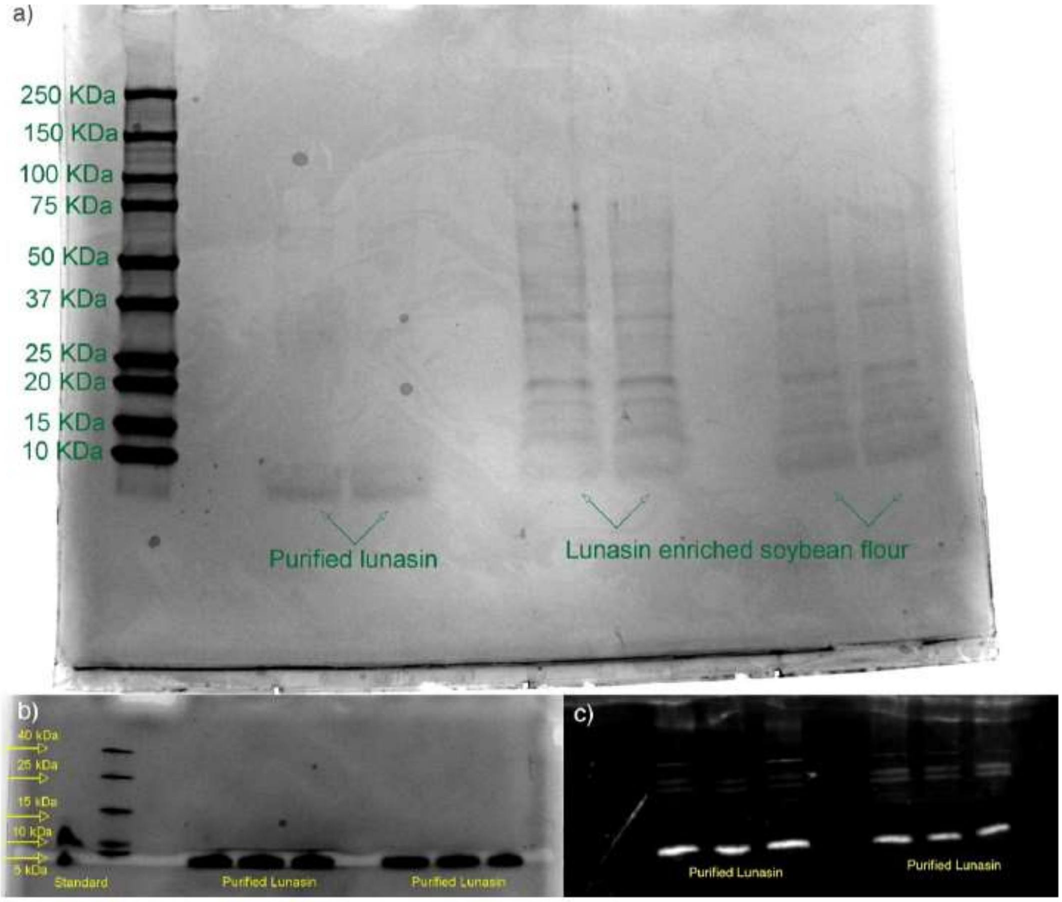

Figure S1. Lunasin purity after extraction. (**a**) High molecular weight gel electrophoresis. (**b**) Low molecular weight gel electrophoresis. (**c**) Western blot.
